# Association of gravity drainage and complications following Whipple: an analysis of the ACS-NSQIP targeted database

**DOI:** 10.1186/s12957-021-02227-0

**Published:** 2021-04-14

**Authors:** Bradley R. Hall, Zachary H. Egr, Robert W. Krell, James C. Padussis, Valerie K. Shostrom, Chandrakanth Are, Bradley N. Reames

**Affiliations:** 1 Division of Surgical Oncology, Department of Surgery, University of Nebraska Medical Center, 986880 Nebraska Medical Center, Omaha, NE 68198 USA; 2 College of Medicine, University of Nebraska, Omaha, NE USA; 3 Department of Surgery, Brooke Army Medical Center, Fort Sam Houston, TX USA; 4 College of Public Health, Department of Biostatistics, University of Nebraska Medical Center, Omaha, NE USA

**Keywords:** Pancreatic ductal adenocarcinoma, Drain, Suction, Gravity, Morbidity

## Abstract

**Background:**

The optimal type of operative drainage following pancreaticoduodenectomy (PD) remains unclear. Our objective is to investigate risk associated with closed drainage techniques (passive [gravity] vs. suction) after PD.

**Methods:**

We assessed operative drainage techniques utilized in patients undergoing PD in the ACS-NSQIP pancreas-targeted database from 2016 to 2018. Using multivariable logistic regression to adjust for characteristics of the patient, procedure, and pancreas, we examined the association between use of gravity drainage and postoperative outcomes.

**Results:**

We identified 9665 patients with drains following PD from 2016 to 2018, of which 12.7% received gravity drainage. 61.0% had a diagnosis of adenocarcinoma or pancreatitis, 26.5% had a duct <3 mm, and 43.5% had a soft or intermediate gland. After multivariable adjustment, gravity drainage was associated with decreased rates of postoperative pancreatic fistula (odds ratio [OR] 0.779, 95% confidence interval [CI] 0.653–0.930, *p*=0.006), delayed gastric emptying (OR 0.830, 95% CI 0.693–0.988, *p*=0.036), superficial SSI (OR 0.741, 95% CI 0.572–0.959, *p*=0.023), organ space SSI (OR 0.791, 95% CI 0.658–0.951, *p*=0.012), and readmission (OR 0.807, 95% CI 0.679–0.958, *p*=0.014) following PD.

**Conclusions:**

Gravity drainage is independently associated with decreased rates of CR-POPF, DGE, SSI, and readmission following PD. Additional prospective research is necessary to better understand the preferred drainage technique following PD.

## Introduction

The approach to abdominal drainage after a major abdominal surgery has evolved in recent decades. While many procedures employ selective drainage, drains are still commonly utilized after pancreatectomy [1–6]. Reasons commonly given for routine pancreatic drainage are early diagnosis of and management of pancreatic fistula, which is a potentially devastating complication [7]. Whether operative drains help prevent or manage complications remains debated. While some clinical trials addressing this question showed similar complications and mortality regardless of closed-suction drain usage, others were stopped early after showing increased mortality in patients without drains [8–10]. As a result, drain placement in pancreatectomy remains discretionary and subject to institutional and surgeon preference.

Regardless of the decision to utilize operative drains, the preferred method of closed drainage following pancreatectomy is poorly understood. Two of the most commonly utilized methods are suction and gravity drainage. The most commonly cited reason for using gravity drainage is avoiding suction near the pancreaticoenteric anastomosis, which may cause or contribute to the development of postoperative pancreatic fistula (POPF) [11–14]. Supporting this argument are institutional series associating higher leak rates with prolonged suction drainage and decreased leak rates associated with gravity drainage [12, 13]. In contrast, recent publications have not demonstrated differences in POPF rates between closed-suction and gravity drains in mixed cohorts of pancreatectomy [15] or among a small cohort of pancreaticoduodenectomy (PD) alone [16].

A better understanding of the relationship between drain suction and complications following PD could inform best practice and possibly improve patient outcomes. Therefore, in this study, we sought to investigate the relationship between type of drainage and procedure-specific complications following PD, using a validated international surgical registry, similar to prior reports. However, as recent studies have been limited by heterogenous cohorts, methodological considerations, or small sample sizes, in this study, we address these limitations by using a rigorous and previously published definition of POPF congruent with the revised International Study Group of Pancreatic Fistula (ISGPS) definition of 2016, and we evaluate a large homogeneous cohort of PD only.

## Material and methods

### Ethics approval and consent to participate

All data within this study was obtained from a registry comprised of de-identified information. As such, it was exempt from review by our University’s Institutional Review Board. Similarly, since no individual patient data was analyzed, individual patient consent was forgone.

### Data source

The American College of Surgeons National Surgical Quality Improvement Program (ACS-NSQIP) is a clinical registry that collects preoperative data and 30-day outcomes for patients undergoing surgical procedures from 713 hospitals around the world, of which 581 are located in the USA [17]. Details regarding data abstraction and validity have been reported elsewhere [18]. Beginning in 2014, the ACS-NSQIP offered procedure-targeted data collection for pancreatectomy procedures for select hospitals [17]. The procedure-targeted dataset includes 100% case capture for pancreatectomy procedures and additional preoperative, intraoperative, and postoperative variables. For this study, we merged the pancreatectomy procedure-targeted dataset from 2016 to 2018 with the contemporaneous ACS-NSQIP Participant User Data File.

### Availability of data and materials

All data analyzed in this study originated from the ACS-NSQIP Participant User Data File. This data may be obtained by requesting the Participant User Data Files from the American College of Surgeons at https://www.facs.org/quality-programs/acs-nsqip.

### Study population and variables

We identified all adults undergoing PD that also received operative drain placement from 2016 to 2018 in ACS-NSQIP participating hospitals with pancreatectomy-targeted data collection using relevant Current Procedural Terminology (CPT) codes. We excluded patients undergoing emergency surgery and patients with open drainage systems or missing data regarding drainage.

Available patient characteristics included age, gender, race, American Society of Anesthesiologists (ASA) class, diabetes, and administration of neoadjuvant chemotherapy or radiation. Operative data included surgical approach (open vs. minimally invasive), wound classification, prophylactic antibiotic usage, wound protector usage, operative duration, pancreatic duct diameter, gland texture, vascular reconstruction, type of pancreaticoenterostomy, red blood cell transfusion on postoperative day (POD) 0, and pancreatic pathology.

### Postoperative outcomes

The primary outcome of this study was clinically relevant POPF (CR-POPF) in accordance with the updated ISGPF definition from 2016 [19]. As ACS-NSQIP does not specifically code pancreatic fistula according to this definition, the variable CR-POPF was created using available registry data as previously described in the literature [20, 21]. In brief, patients defined as having a “pancreatic fistula” or those with a drain amylase >300 U/dL on or after POD 3 were considered to have a CR-POPF if they met any of the following criteria: (1) drain in place for >21 days or >14 days with prolonged hospital stay >21 days; (2) postoperative percutaneous drain placed, or (3) presence of organ space surgical site infection (SSI), reoperation, sepsis, shock, or multisystem organ failure [20, 21].

Secondary outcomes of interest included delayed gastric emptying (DGE), percutaneous drain placement, reoperation, readmission, prolonged length of stay (LOS), mortality, and surgical site compilations including superficial, deep, or organ space SSI; sepsis; and septic shock. Delayed gastric emptying was defined by NSQIP as no oral intake by POD 14 or nasogastric/gastric drainage or replacement after POD 7. Prolonged length of stay was defined as a LOS >75th percentile. Mortality was defined as death within 30 days of surgery or prior to discharge. For all infectious outcomes, a complication was documented if it occurred within 30 days of the operation and was not documented as present on admission.

### Statistical analysis

Student’s *t*-test, Chi-square, or Fisher’s exact tests were used as appropriate to evaluate univariable associations between primary and secondary outcomes and the type of operative drainage (suction vs. gravity). We then assessed the influence of drainage type on CR-POPF and secondary outcomes using multivariable logistic regression, adjusting for patient demographics, comorbidities, and operative differences across groups. Patient and operative characteristics associated with CR-POPF and secondary outcomes were identified using stepwise logistic regression with backwards selection to ensure appropriate model fit. Candidate variables in the model included patient age, gender, race, smoking, diabetes, year of procedure, ASA classification ≤2, wound classification ≥3, neoadjuvant chemotherapy, neoadjuvant radiation, surgical approach, vascular reconstruction, pancreas reconstruction, surgical pathology, pancreatic gland texture and pancreatic duct size, and POD 0 transfusion. For modeling purposes, minimally invasive procedures that converted to open were considered open surgery. For the final analysis and in accordance with previous literature evaluating pancreatic fistula risk [20, 21], surgical pathology was dichotomized as “adenocarcinoma or pancreatitis” or “other,” and pancreatic gland texture was dichotomized as “hard” or “intermediate/soft” [22]. As the estimated blood loss of the operation was not available, associations with this variable were tested using the administration of a red blood cell transfusion on POD 0.

Variables from the stepwise regression model with coefficient *p*-values <0.05 were then used in a second multivariable model including drainage type. In our model assessing drainage on CR-POPF, the variable duct size (<3mm, ≥3mm, or unknown), firm gland texture, surgical pathology, and POD 0 transfusion were forced into the model regardless of significance. To account for missing data, particularly within variables above known to influence the risk of CR-POPF, we performed multiple sensitivity analyses. First, the analysis was performed only using observations with complete data for surgical pathology, gland texture, duct size, and POD 0 transfusion. Second, multiple imputation was used to account for the missing data within each variable. As the results of both analyses were qualitatively identical to the primary analysis, only the results of the primary analysis are presented here. Statistical analyses were performed using SAS v9.4 (SAS Institute Inc., Vary, NC, USA). For all statistical tests, *p* values are two-tailed, and alpha is set at 0.05.

## Results

A total of 9665 PD patients received operative drainage between 2016 and 2018 at ACS-NSQIP targeted pancreatectomy participating hospitals. Demographic, operative, and pathologic characteristics, according to type of drainage, are shown in Table 1. Of 9665 patients with operative drains, 1224 (12.7%) had drains to gravity. Patients with closed-suction drains were more likely to have ASA classification 3 or 4 (78.1% vs. 74.3%, *p*=0.002), received neoadjuvant chemotherapy (22.3%, vs. 18.4%, *p*=0.002) and radiation (9.2% vs. 6.2%, *p*=0.001), underwent more vascular resections (17.3% vs. 13.8%, *p*=0.003), and more frequently had duct-mucosa pancreaticojejunostomy reconstructions (89.1% vs. 86.8%, *p*=0.015).

**Table 1:** Patient characteristics and operative details

Variable	Suction (*N*=8441)		Gravity (*N*=1224)		***p***-value
	Number	Percent	Number	Percent	
Age (median, [IQR])	67 [59, 73]		67 [58, 73]		**0.048**
Female	3891	46.1%	598	48.9%	0.070
Race					0.293
White	6284	74.4%	894	73.0%	
Unknown/other	2157	25.6%	330	27.0%	
Smoking					**0.048**
Yes	1461	17.3%	184	15.0%	
No	6980	82.7%	1040	85.0%	
Diabetes					0.146
Yes	2207	26.2%	344	28.1%	
No	6234	73.8%	880	71.2%	
Operation year					**<0.001**
2016	2581	30.6%	498	40.7%	
2017	2853	33.8%	378	30.9%	
2018	3007	35.6%	348	28.4%	
ASA class					**0.002**
1 or 2	1845	21.9%	315	25.7%	
3 or 4	6596	78.1%	909	74.3%	
Wound class					**<0.001**
1 or 2	6802	80.6%	1038	84.8%	
3 or 4	1639	19.4%	186	15.2%	
Neoadjuvant chemotherapy					**0.002**
Yes	1884	22.3%	225	18.4%	
No	6557	77.7%	999	81.6%	
Neoadjuvant radiation therapy					**0.001**
Yes	777	9.2%	76	6.2%	
No	7664	90.8%	1148	93.8%	
Approach (combined categories)					**0.013**
Open	7925	93.9%	1171	95.7%	
Minimally invasive	516	6.1%	53	4.3%	
Pancreatic duct diameter					**<0.001**
<3 mm	2254	26.7%	324	26.5%	
≥3 mm	4697	55.6%	615	50.2%	
Unknown	1490	17.7%	285	23.3%	
Pancreatic gland texture					**<0.001**
Soft/intermediate	3890	46.1%	533	43.5%	
Hard	2797	33.1%	336	27.5%	
Unknown	1754	20.8%	335	27.4%	
Pancreatic pathology					0.264
Adenocarcinoma or pancreatitis	5010	59.4%	747	61.0%	
Other or unknown	3431	40.6%	477	39.0%	
Transfusion on POD0					**0.001**
Yes	1169	13.8%	127	12.1%	
No	7272	86.2%	1097	83.9%	
Pathologic detail					--
Pancreatic adenocarcinoma	4614	55.4%	687	56.9%	
Ampullary carcinoma	683	8.2%	88	7.3%	
Duodenal carcinoma	255	3.1%	32	2.7%	
Neuroendocrine	550	6.6%	88	7.3%	
IPMN-invasive	188	2.3%	17	1.4%	
Distal cholangiocarcinoma	273	3.3%	24	2.0%	
Malignant other	419	5.0%	50	4.1%	
Chronic pancreatitis	288	3.5%	51	4.2%	
IPMN-noninvasive	534	6.4%	96	7.9%	
Mucinous cystic neoplasm	59	0.7%	5	0.4%	
Serous cystadenoma	78	0.9%	14	1.2%	
Solid pseudopapillary neoplasm	43	0.5%	7	0.6%	
Benign other	342	4.1%	49	4.1%	
Pancreatic reconstruction					**0.015**
Pancreaticojejunal duct-to-mucosal	7521	89.1%	1062	86.8%	
Pancreaticojejunal invagination or pancreaticogastrostomy	920	10.9%	162	13.2%	
Vascular reconstruction (any)	1457	17.3%	169	13.8%	**0.003**
T stage					--
T0/Tis	110	1.3%	8	0.7%	
T1	899	10.9%	125	10.4%	
T2	1777	21.5%	272	22.7%	
T3	3629	43.8%	502	41.8%	
T4	381	4.6%	47	3.9%	
Tx/unknown	1484	17.9%	246	20.5%	
N stage					--
N0	2689	32.7%	369	31.0%	
N1	4029	49.1%	573	48.2%	
Nx/unknown	1493	18.2%	248	20.8%	
M stage					--
M0/Mx	5381	70.2%	615	61.6%	
M1	145	1.9%	28	2.8%	
Unknown	2136	27.9%	356	35.6%	

Results from the univariable analysis of postoperative drain management and outcomes are displayed in Table 2. Notably, rates of CR-POPF were more common in the suction group as compared to the gravity group (16.5% vs. 13.7%, *p*=0.015). SSIs were more frequent in the closed-suction drain group, particularly superficial SSI (7.3% vs. 5.6%, *p*=0.033) and organ space SSI (14.9% vs. 12.4%, *p*=0.022). Unplanned readmission (17.6% vs. 14.1%, *p*=0.008) and DGE (16.6% vs. 14.0%, *p*=0.019) were also more common in the closed-suction group. Length of stay was similar between groups. Notably, no significant differences in rates of percutaneous drain placement, reoperation, sepsis, or mortality were observed.

**Table 2:** Univariable associations between drain management and outcomes for the entire cohort

Outcome	Suction (*N*=8441)	Gravity (*N*=1224)	***p***-value
CR-POPF	1389 (16.5%)	168 (13.7%)	**0.015**
Mortality	158 (1.9%)	14 (1.1%)	0.072
Surgical site infection (SSI)			
Superficial	617 (7.3%)	69 (5.6%)	**0.033**
Deep incisional	83 (1.0%)	7 (0.6%)	0.161
Organ-space	1257 (14.9%)	152 (12.4%)	**0.022**
Any SSI	1801 (21.3%)	210 (17.2%)	**0.001**
Sepsis	570 (6.7%)	72 (5.9%)	0.253
Reoperation	475 (5.6%)	54 (4.4%)	0.081
Unplanned readmission	1486 (17.6%)	173 (14.1%)	**0.008**
Delayed gastric emptying	1403 (16.6%)	171 (14.0%)	**0.019**
Percutaneous drain placement	1047 (12.4%)	153 (12.5%)	0.924
Prolonged length of stay^a^	2318 (27.8%)	313 (25.9%)	0.165

^a^Greater than or equal to 12 days

*CR-POPF* clinically relevant postoperative pancreatic fistula, *SSI* surgical site infection

Table 3 displays multivariable associations between patient and disease characteristics and the primary outcome, CR-POPF, in the final logistic regression model. After adjusting for relevant patient and treatment characteristics, including surgical pathology, pancreatic gland texture and duct size, and transfusion on POD 0, use of closed-suction drainage systems is independently associated with risk of CR-POPF (adjusted odds ratio 1.283, 95% confidence interval 1.075–1.532, *p*=0.006). Other characteristics significantly associated with risk of CR-POPF in the model include small pancreatic duct size, soft/intermediate pancreatic gland texture, surgical pathology other than PDAC or pancreatitis, gender, white race, diabetes, neoadjuvant chemotherapy and radiation, minimally invasive surgery, and BMI.

**Table 3:** Multivariable associations between drain management and CR-POPF

Variable	AOR	95% CI		***p***-value
		Lower	Upper	
Closed-suction drainage	1.283	1.075	1.532	**0.0058**
Pancreatic duct size				
> 3 mm	Ref	Ref	Ref	Ref
< 3 mm	1.366	1.199	1.555	**<.0001**
Unknown	1.277	1.068	1.527	**0.007**
Pancreatic gland texture				
Hard	Ref	Ref	Ref	Ref
Soft/intermediate	2.596	2.212	3.046	**<.0001**
Unknown	1.952	1.598	2.384	**<.0001**
Pathology other than PDAC or pancreatitis	1.372	1.217	1.548	**<.0001**
POD 0 transfusion	1.113	0.941	1.316	0.2121
Female	0.697	0.622	0.781	**<.0001**
White race	1.145	1.010	1.299	**0.0345**
Diabetes	0.779	0.681	0.892	**0.0003**
Neoadjuvant chemotherapy	0.751	0.623	0.906	**0.0027**
Neoadjuvant radiation	0.707	0.527	0.949	**0.0211**
Minimally invasive surgery	0.615	0.471	0.805	**0.0004**
Pancreatic reconstruction other than duct to mucosa pancreaticojejunostomy	1.122	0.947	1.329	0.1829
BMI	1.026	1.017	1.036	**<.0001**

*CR-POPF* clinically relevant postoperative pancreatic fistula, *PDAC* pancreatic ductal adenocarcinoma, *POD* postoperative day

**Fig. 1:**
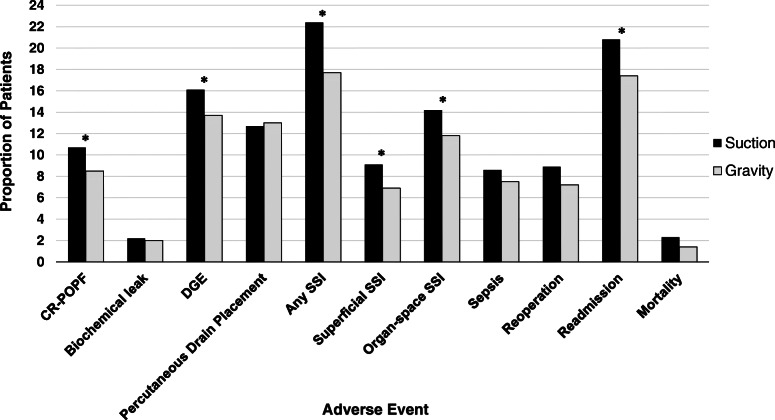
Adjusted rates of postoperative outcomes following pancreaticoduodenectomy. (Significant differences with *p*-values <0.05 are indicated with an asterisk; CR-POPF, clinically relevant postoperative pancreatic fistula; DGE, delayed gastric emptying; SSI, surgical site infection)

The adjusted rates of postoperative outcomes for suction and gravity drainage are displayed in Fig. 1, while the corresponding odds ratios for gravity drainage are shown in Table 4. After adjusting for patient and operative risk factors, the rates of CR-POPF (10.7% vs. 8.5%, *p*=0.006), delayed gastric emptying (16.1% vs. 13.7%, *p*=0.036), any SSI (22.4% vs. 17.7%, *p*<0.001), superficial SSI (9.1% vs. 6.9%, *p*=0.023), organ space SSI (14.2 vs. 11.8, *p*=0.012), and readmission (20.8% vs. 17.4%, *p*=0.014) were lower in the gravity drainage group as compared to the closed-suction group. No adverse outcomes were more common in the gravity cohort. The odds of experiencing CR-POPF were statistically significantly lower in patients receiving gravity drainage (adjusted odds ratio [AOR] 0.779, 95% confidence interval [CI] 0.653–0.930), as were the odds of DGE (AOR 0.830, 95% CI 0.693–0.988), any SSI (AOR 0.741, 95% CI 0.631–0.870), superficial SSI (AR 0.741, 95% CI 0.572–0.959), organ space SSI (AR 0.791, 95% CI 0.658–0.951), and readmission (AOR 0.807, 95% CI 0.679–0.958).

**Table 4:** Adjusted odds ratios (AOR) for gravity drainage and outcomes

Outcome	AOR	95% CI		***p***-value
		Lower	Upper	
CR-POPF	0.779	0.653	0.930	**0.0058**
Biochemical leak	0.919	0.693	1.219	0.556
Delayed gastric emptying	0.830	0.693	0.988	**0.036**
Percutaneous drain placement	1.024	0.851	1.233	0.800
Any surgical site infection (SSI)	0.741	0.631	0.870	**<0.001**
Superficial SSI	0.741	0.572	0.959	**0.023**
Organ-space SSI	0.791	0.658	0.951	**0.012**
Sepsis	0.956	0.664	1.105	0.233
Reoperation	0.794	0.594	1.062	0.120
Readmission	0.807	0.679	0.958	**0.014**
Mortality	0.640	0.368	1.112	0.113

*CR-POPF* clinically relevant postoperative pancreatic fistula, *SSI* surgical site infection

## Discussion

This analysis of the ACS-NSQIP procedure-targeted database demonstrates that among patients with operative drain placement after PD, closed-suction drainage is independently associated with higher rates of CR-POPF (10.7% vs. 8.5%), DGE (16.1 vs. 13.7%), SSI (22.4% vs. 17.7%), and readmission (20.8% vs. 17.4%). Given the sparse literature on this subject and the mechanistic plausibility of the association, the results of this study raise important questions about the type of operative drainage utilized following PD and warrant further investigation.

Few studies have been published comparing gravity and closed-suction drainage in pancreas surgery, and much of the literature to date suffers from substantial limitations. In the single-institution retrospective study by Schmidt and colleagues, gravity drainage was associated with lower rates of POPF (14% vs. 3%), but also correlated with higher volume surgeons, raising concerns about gravity drainage acting as a surrogate for procedure volume [13]. Though multiple recently published studies suggest no differences in outcomes between suction or gravity closed-drainage systems [15, 16, 23], each of these studies has significant limitations that warrant consideration. Previous randomized trials evaluating drainage type have been limited by small sample size or showed rates of CR-POPF higher than those typically seen in US hospitals [23]. The study by Kone et al. included only the 2016–2017 ACS-NSQIP pancreas-targeted database and analyzed both PD and distal pancreatectomy together. In addition, the authors used the ACS-NSQIP definition of CR-POPF, which is not congruent with the updated 2016 ISGPS definition [20], and the use of propensity score matching resulted in the loss of >10% of their sample size. As previous literature clearly demonstrates that POPF risk differs between PD and distal pancreatectomy [9, 10], studies of pancreatectomy investigating CR-POPF should be performed on homogeneous populations to minimize confounding of the results. Lemke and colleagues, on the other hand, used a more rigorous definition of CR-POPF and a more homogenous population of PD patients. However, the power of this study is severely limited by its small sample size, as their final multivariable analysis only included 1787 patients, and use of coarsened exact matching further reduced their final cohort to 268 patients. Not unexpectedly, no association between drainage technique and CR-POPF was observed.

Our study addresses the limitations of these previous studies by using 3 years of a large, validated, international surgical registry to increase sample size; studies a selected population of PD procedures only; and utilizes a rigorous previously published definition of CR-POPF congruent with the ISGPS update of 2016 [20, 21]. Similar to recent studies above, the results herein are consistent in demonstrating a small decrease in rates of CR-POPF associated with use of gravity drainage when compared to closed-suction drainage. However, in contrast recent work, the findings of this study (with a larger sample and analyses maximizing use of all available patients) *do* suggest a statistically significant relationship between gravity drainage and consistently lower rates of CR-POPF, and multiple other related complications, following PD.

In pancreas surgery, much of the literature evaluating operative drainage is focused on addressing whether drains are necessary at all, and results remain conflicted. In one of the earliest trials addressing this issue, Conlon et al. found no differences in overall morbidity or mortality in patients undergoing PD or distal pancreatectomy regardless of drain usage. In that study, POPF rates were higher in the drained group, suggesting either a detection bias or promotion of fistula formation in drained patients [8]. These results were later supported by findings from Witzigmann and colleagues in the pancreatic drainage (PANDRA) trial, but contradicted by Van Buren et al., who found higher mortality rates in PD patients without operative drains [9, 24, 25]. However, these studies had multiple inherent limitations. Similar to above, considering that the risk associated with operative drainage is procedure-dependent, the inclusion of all partial pancreatectomy patients by Conlon and colleagues may have influenced results [8–10]. The PANDRA trial also suffered from protocol violations and randomization issues [24]. In the later study, the definition of POPF differed from that set forth by the ISGPS, and operative drains were used in approximately 15% of cases performed by surgeons who were classified as routinely omitting drains [22, 25]. Given the lack of consensus in the literature and the potential for severe morbidity from an uncontrolled pancreatic leak, operative drains are placed in the majority of pancreatectomy cases [7].

Considering that operative drains remain heavily utilized, recent literature has focused on selecting patients for drain omission or identifying patients in whom drains can be safely removed early [22, 26, 27]. Several risk scores are available to stratify patients according to risk of CR-POPF, and many surgeons use these scores to select patients for drain omission [22, 26, 27]. Similarly, several postoperative drain management algorithms employing drain amylase levels are routinely used to identify patients in whom drains can be safely removed [28, 29]. However, neither the fistula risk calculations nor drain management algorithms published to date account for drainage type. Our study shows a significant association between gravity drainage and decreased CR-POPF after PD. These results suggest that the type of drainage should be considered in the management of PD patients with operative drains.

The mechanistic plausibility of an association between closed-suction drainage and increased rates of CR-POPF is clear and supported by several studies in the literature [11–13]. Whitson and colleagues also measured the force of several types of operative drains and estimated that the pressure applied from a 100-mL Jackson Pratt bulbs was approximately −125 mmHg [14]. Such force may interfere with the normal healing process in an already tenuous pancreaticoenteric anastomosis. As the development of biochemical leak and CR-POPF are known risk-factors for other post-pancreatectomy complications, such as superficial SSI, organ-space SSI, delayed gastric emptying, and others (readmission), it would be expected that, in a true observation of the world, an analysis reporting an association between gravity drainage and decreased rates of CR-POPF would also report associations with decreased rates of other common downstream complications [30–32]. At the same time, when considering the proposed mechanism, it is not surprising that this analysis failed to reveal an association between drainage method and less frequent, more severe complications (e.g., reoperation, organ failure, or mortality) associated with grade C POPF. Grade C POPF often occur secondary to a major anastomotic failure or disruption that results in a large volume leak and a severe systemic inflammatory response. This type of complication is commonly technical in nature and is unlikely to be influenced by the method of drainage employed.

This study has several limitations. First, the ACS-NSQIP procedure-targeted database lacks surgeon- and institution-specific variables that may further influence CR-POPF rates after PD, and as with any clinical registry, there is no doubt of residual confounding in these assessments. For example, it is likely that many individual surgeons do not alternate the type of drainage employed. As a result, the use of gravity drainage could instead represent other unmeasured surgeon- or institution-specific variables (such as volume, technical skill, practice patterns, or culture) known to be associated with outcomes. However, the ACS-NSQIP procedure-targeted database is currently the largest and most reliable contemporaneous clinical registry available to answer this research question, and the multivariable models used herein included patient, pancreas, and procedure-specific variables (components of the FRS) widely accepted to influence risk of POPF. A second and similar limitation of this study is that results may not be generalizable to institutions not participating in ACS-NSQIP. However, the institutional cohort of NSQIP is diverse and includes a broad range of hospitals: critical access, community, and tertiary academic referral centers, urban and rural, for-profit and not-for-profit. Finally, this is a retrospective analysis, and causation cannot be inferred from our results. While the associations reported herein should only be considered hypothesis generating, the consistency of the associations and the biologic plausibility of the mechanism warrant further investigation.

This is one of the largest studies to date addressing the question of preferred type of operative drainage following PD. Closed-suction drainage was consistently associated with higher rates of multiple pancreatectomy-specific complications, including the primary outcome, CR-POPF, as well as multiple associated downstream complications, such as superficial SSI, organ-space SSI, and DGE. In the context of disparate results from clinical trial data, these results challenge the prevailing practice of closed-suction drainage. If closed-suction drainage is found to contribute to formation of POPF, the results could have important implications for operations beyond PD that involve delicate or technically difficult anastomoses. Additional prospective and (ideally) randomized research is needed to separately address this question both for patients undergoing PD and those undergoing distal pancreatectomy and splenectomy.

## Data Availability

This registry database is available to all participating members of the ACS NSQIP collaborative and can be requested here https://www.facs.org/quality-programs/acs-nsqip.
